# Impact of the COVID-19 Pandemic on Corneal Transplantation: A Report From the Italian Association of Eye Banks

**DOI:** 10.3389/fmed.2022.844601

**Published:** 2022-03-22

**Authors:** Rita Mencucci, Michela Cennamo, Diego Ponzin, Federico Genzano Besso, Giulio Pocobelli, Matilde Buzzi, Carlo Nucci, Francesco Aiello

**Affiliations:** ^1^Department of Neurosciences, Psychology, Pharmacology and Child Health, Eye Clinic, University of Florence, Florence, Italy; ^2^The Veneto Eye Bank Foundation, Venice, Italy; ^3^Eye Bank of Piedmont, SSD Tissue Banks and Biorepository, Città della Salute e della Scienza, Turin, Italy; ^4^Ophthalmology Unit, Department of Experimental Medicine, University of Rome “Tor Vergata”, Rome, Italy

**Keywords:** SARS-CoV-2, eye bank guidelines, corneal transplant, eye donor screening, endokeratoplasty

## Abstract

**Purpose:**

To analyze the impact of COVID-19 on Italian corneal transplantation from March-2020 to February 2021 compared to the same timeframe of the 2 previous years, in order to identify potential consequences of a global pandemic on corneal procurement and transplantation services during this time.

**Methods:**

This national, multicentric, retrospective cohort study evaluated data collected from 12 (100%) Italian eye banks from March 2020 to February 2021 (Group A). The number of tissues collected, distributed and discarded were compared with the same time-frame of the 2 previous years: 2019 and 2018 (group B and C, respectively). The different type of transplants performed were reported. Data were analyzed using a non-parametric Friedman test.

**Results:**

Corneal procurement and the percentage of distributed tissues reduced in 2020 by more than 30 and 15%, respectively, compared to the 2 previous years. During the pandemic corneal transplant surgery showed only a modest drop: the number of the penetrating keratoplasties (PKs) and the anterior lamellar keratoplasties (ALKs) decreased by about 30 and 20% in comparison with groups B and C, respectively; between the Endothelial Keratoplasties (EKs), the Descemet membrane endothelial keratoplasty (DMEK) increased slightly from March 2020 to February 2021.

**Conclusions:**

Italy was one of the first countries most affected by the outbreak of COVID-19, and the Italian government adopted severe measures to limit viral transmission. The pandemic generated several implications in corneal transplant activity during the first lockdown. Then an efficacious reaction with constant, vigorous work led to a resumption of transplant surgery to a near-normal standard. The increase of EKs, despite the pandemic, is a sign that the advance in corneal transplantation has gone ahead and it continues to evolve.

## Introduction

The difficult scenario, experienced during the first COVID-19 pandemic wave, and that negatively impacted the National Health System by diverting resources to contain this infection, influenced the “second wave” which started around 1st October 2020. The lessons learned from the first wave led to a less confused and more organized access path to intensive care units (1).

COVID-19 has become a constant threat for solid organ transplantation; as a result, the pandemic has generated significant challenges to Italian procurement and transplantation programs. It has been recorded a substantial decrease during the most critical weeks of the early COVID-19 spread, with a 25% reduction in total donations at a national level, partly due to the unknown relationship between the virus and tissues (2).

In Italy, after an initial period of uncertainty relating to the risk of transmission, corneal transplantation activity has been encouraged by the national regulatory authorities and Italian corneal scientific societies, in order to avoid tissue waste. A previous retrospective study comparing the Italian Eye Bank activity during the lockdown period with the same timeframe of 2018 and 2019 has shown a dramatic reduction in corneal transplant activity, resulting in a significant waste of resources (3).

In this study, we analyzed the impact of COVID-19 on Italian corneal transplantation in the whole year “annus horribilis” from March 2020 to February 2021. We therefore compared the aforementioned period with the same timeframe of the two previous years (2018 and 2019), in order to describe the effects of a global pandemic on Italian corneal procurement and transplantation services during this time.

Our study aimed to analyze the clinical relevance and the potential consequences of the COVID-19 pandemic on corneal transplant activity.

## Materials and Methods

In this national multicentric, retrospective cohort study, we analyzed data from 12 (100%) Italian eye banks, collected by the Italian Eye Bank Society “Societa‘ Italiana Banche degli Occhi” (SIBO), in order to report the eye bank activity between March 2020 and February 2021, comparing it with two previous years. To simplify data reporting we classified the three main different time-frames into 3 groups as follows: Group A: from March 2020 to February 2021; Group B: from March 2019 to February 2020; Group C: from March 2018 to February 2019.

We compared the number of corneas collected and distributed by the eye banks after the completion of the screening tissue process. The decrease in the number of corneas procured and distributed was calculated as a percentage of the average. We also reported the number of wasted corneas (returned back and expired).

Finally, we measured the impact of the COVID-19 pandemic on corneal transplant surgery in Italy, comparing the type of transplants performed: penetrating keratoplasty (PK), anterior lamellar keratoplasty (ALK), Descemet stripping automated endothelial keratoplasty (DSAEK), Descemet membrane endothelial keratoplasty (DMEK) and emergency procedures (EP).

## Statistical Analysis

Data were recorded and analyzed using Microsoft Excel (2021). The mean number of corneas collected, and those discarded, from March 2020 to February 2021 was compared with the previous 2 years using a non-parametric Friedman test (Stata/IC 16, StataCorp, USA). Statistical significance was defined as a *p* < 0.05. Data are resumed in Table 1.

**Table 1:** Data summary from Italian Eye Bank Associations.

	**Group C**	**Group B**	**Group A**
N° of collected tissues	17,099	17,930	11,241
N° of distributed tissues	7,074	7,266	5,928
N° PK	3,403	3,198	2,294
N° DALK	675	778	587
N° DSAEK	2,437	2,619	2,431
N° DMEK	559	671	616
N° EP	315	338	424
N° of discarded tissues	857	847	940
N° of COVID-19 + tissues	0	15	151
N° of canceled procedures	127	145	208
N° of expired tissues	623	591	436
N° of requested albeit not transplanted corneas	107	96	42

*PK, Penetrating keratoplasty; ALK, anterior lamellar keratoplasty; DSAEK, Descemet stripping automated endothelial keratoplasty; DMEK, Descemet membrane endothelial keratoplasty; EP, Emergency procedures*.

*Group A: March 2020–February 2021*.

*Group B: March 2019–February 2020*.

*Group C: March 2018–February 2019*.

## Results

### Italian Eye Bank Procurement and Distribution Activity

**Figure 1:**
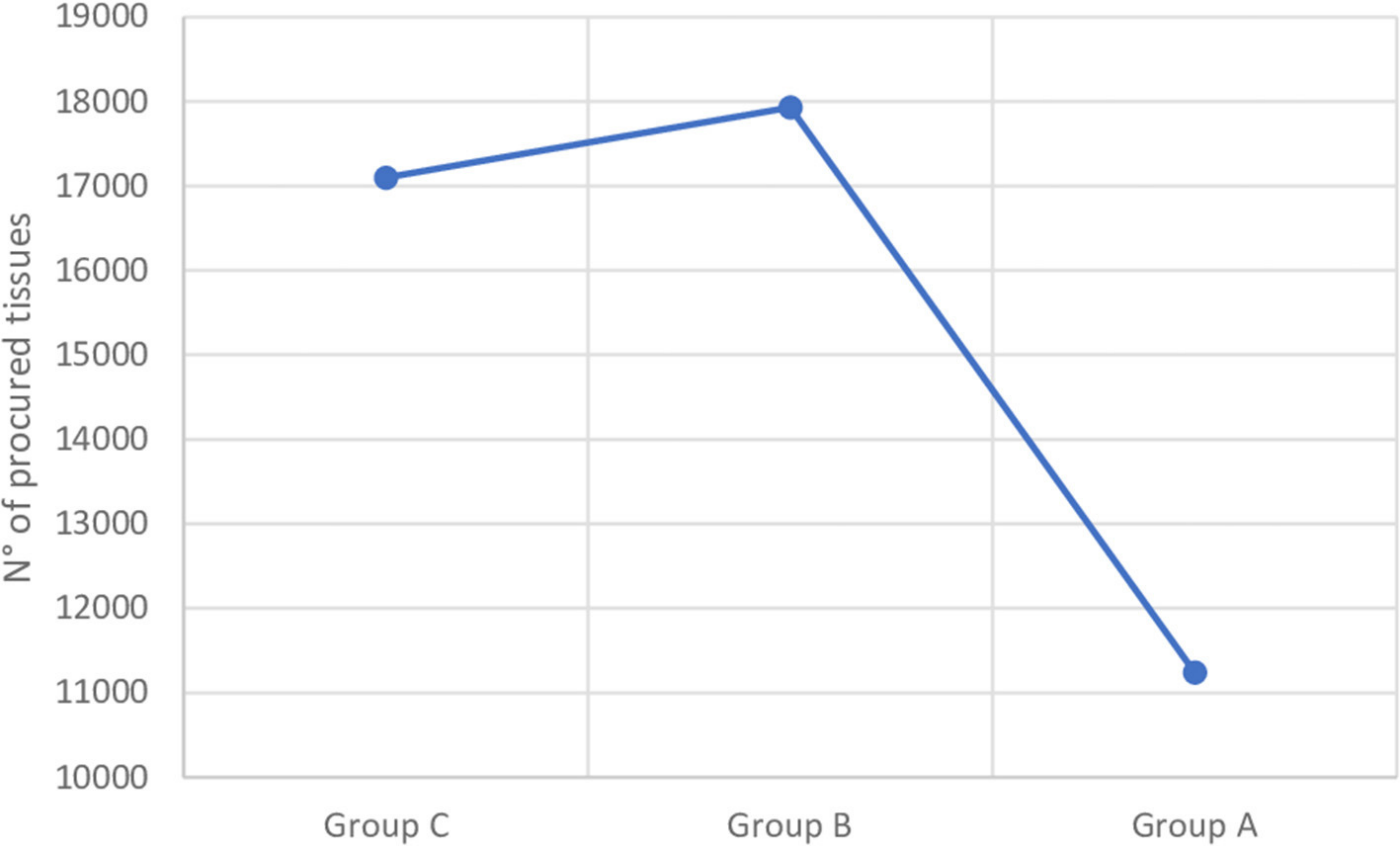
Corneal procurement trend of Italian Association of Eye Banks. Group A, March 2020–February 2021; Group B, March 2019–February 2020; Group C, March 2018–February 2019.

From the 1st March 2020 to the 28th February 2021, 11,241 donor corneas were collected and harvested. Compared to the previous 2 years the number of corneas in Group A appears significantly reduced (Group A: 11,241; Group B: 17,930, (−37.3%); Group C: 17099, (−34.4%); *p*= 0.0009) (Figure 1).

**Figure 2:**
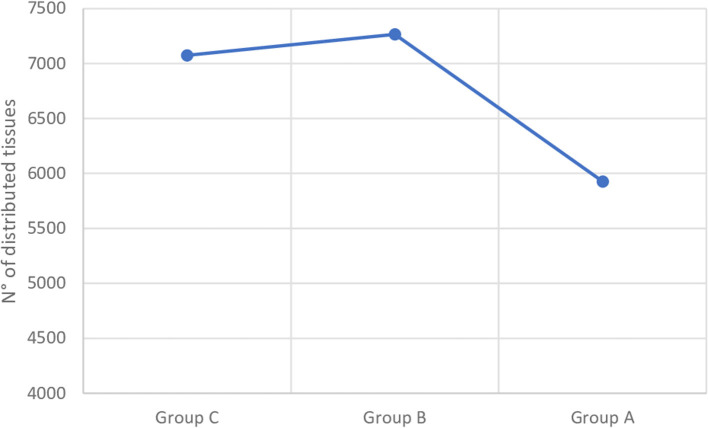
Corneal distribution trend of Italian Association of Eye Banks. Group A, March 2020–February 2021; Group B, March 2019–February 2020; Group C, March 2018–February 2019.

Moreover, we noticed a substantial reduction in the number of distributed tissues when comparing Group A data with the previous 2 years (Group B: −16.2%; Group C: −18.4%) (*p* = 0.0009) (Figure 2).

### Discarded Tissues

**Figure 3:**
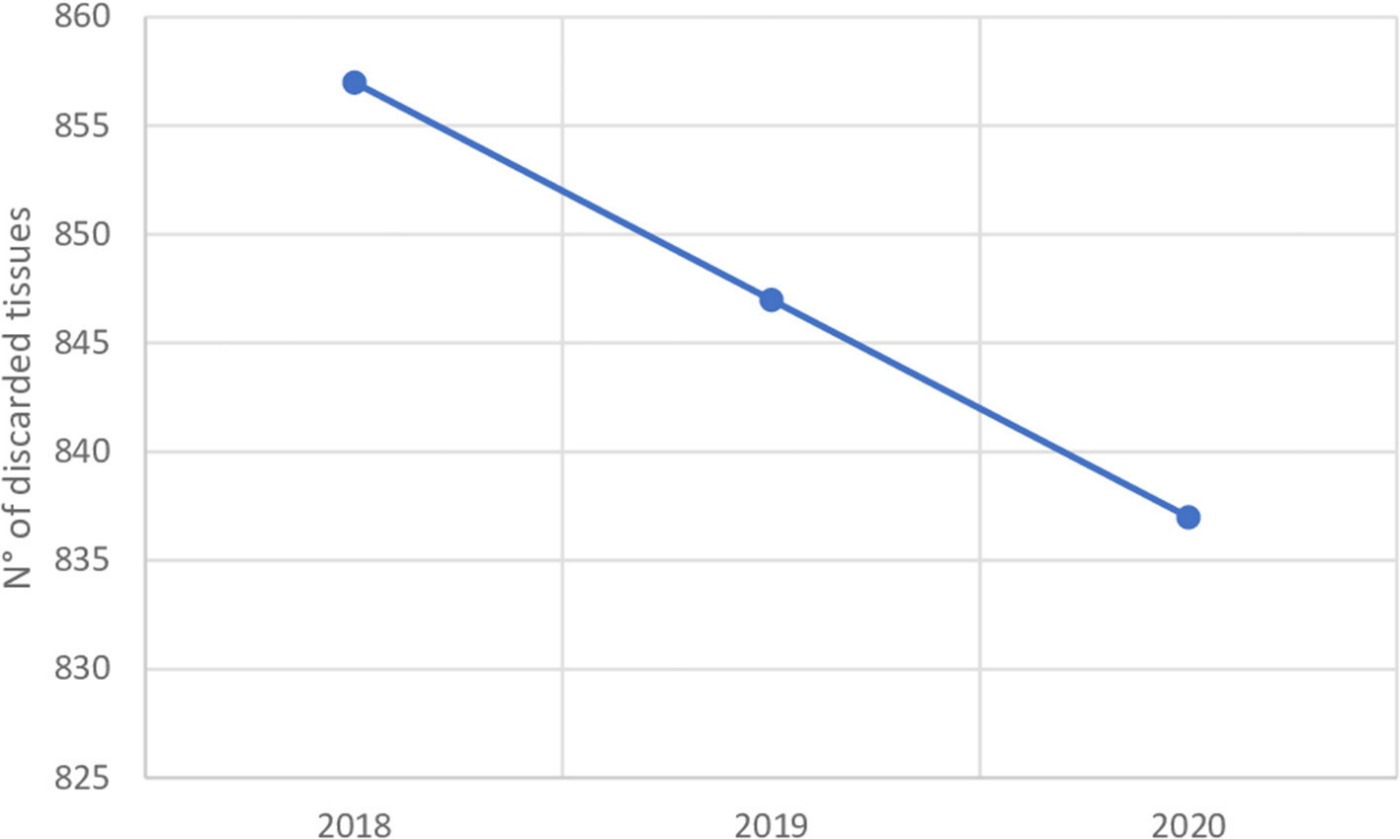
Discarded corneal tissue trend of Italian Association of Eye Banks. Group A, March 2020–February 2021; Group B, March 2019–February 2020; Group C, March 2018–February 2019.

The number of total discarded corneas declined from 857 in Group C to 847 in Group B (−1.17%) and to 837 in Group A (−2.33%%) (*p* = 0.008) (Figure 3).

Among the discharged tissues in Group A, 151 (18%) were dismissed because considered at risk for SARS-CoV-2 contamination. Nonetheless, the percentage of discarded tissues due to a suspicion of SARS-CoV-2 contamination, over the total number of retrieved corneas in Group A, accounted for 1.34%.

Notably, in Group A, 208 surgical procedures were canceled resulting in a 63.8% and 43.5% increase of canceled procedure compared to Group C and Group B, respectively (*p* = 0.003). Nonetheless, the number of expired tissues decreased from 623 in Group C to 591 in Group B and 436 in Group A (−5.1 and −30% respectively) (*p* = 0.001) probably due to a lower number of procured tissues. Similarly, the number of corneas requested, albeit not used by surgeons, declined from 107 to 96 (−10.28%) and to 42 (−60.75%), in Group C, Group B and Group A, respectively (*p* = 0.002).

### Pandemic Impact on Corneal Transplant Surgery

We observed different trends when analyzing the different subtypes of corneal grafts. In particular, the number of PKs significantly decreased from 3,403 in Group C and 3,198 in Group B to 2,294 in Group A (*p* = 0.0006).

**Figure 4:**
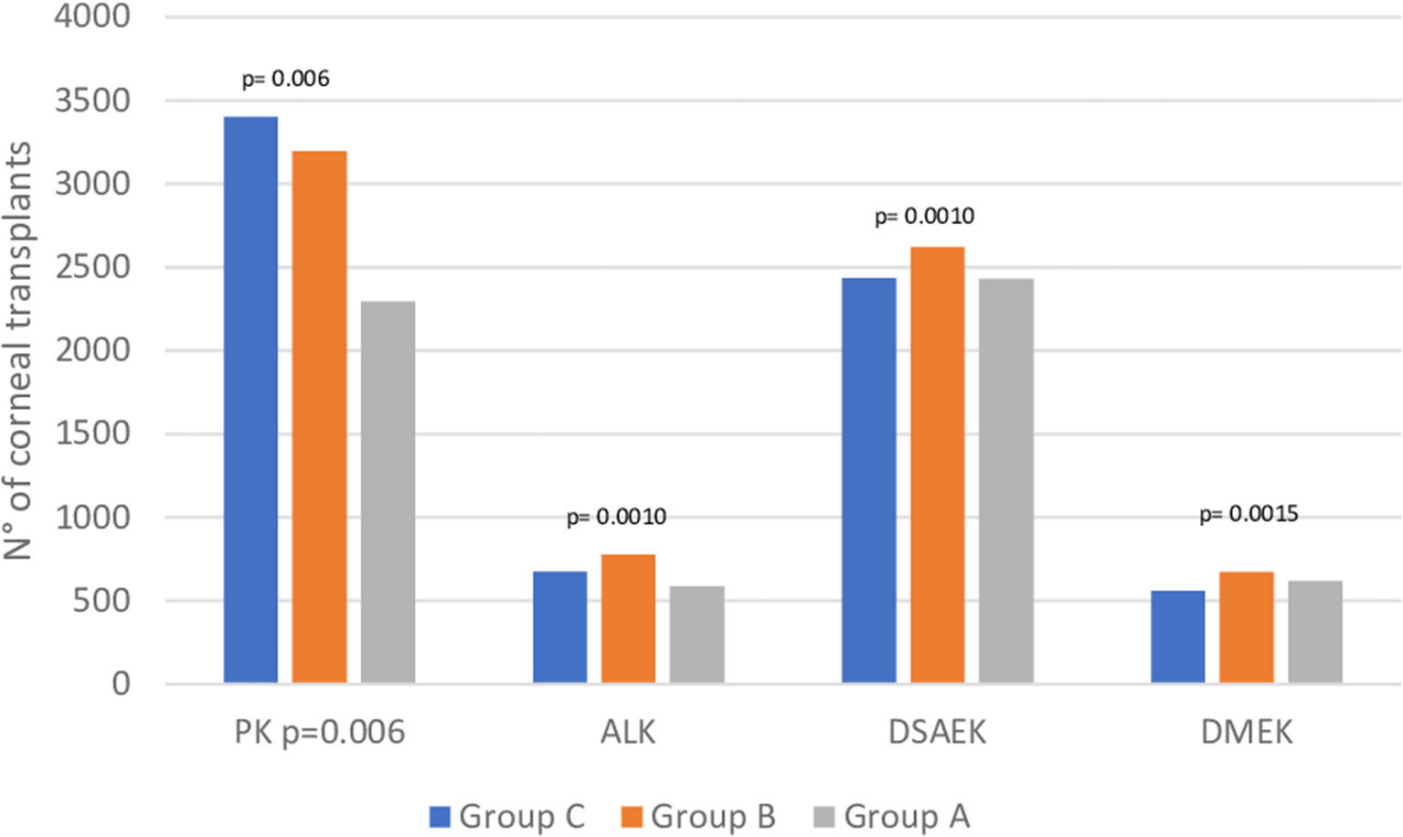
Corneal transplant activity. PK, Penetrating keratoplasty; ALK, Anterior Lamellar Keratoplasty; DSAEK, Descemet Stripping Automated Endothelial Keratoplasty; DMEK, Descemet Membrane Endothelial Keratoplasty. Group A, March 2020–February 202; Group B, March 2019–February 2020; Group C, March 2018–February 2019.

Similarly, the ALK numbers changed from 675 and 778 to 587 in Group C, Group B and Group A, respectively (*p* = 0.001). Interestingly, the endothelial keratoplasties were only partially affected, with 2,437 procedures performed in Group C, 2,619 in Group B and 2,431 in Group A. Nonetheless, the number of DMEKs increased during the study period, from 559 in Group C to 671 (+20%) and 616 (+10.2%) in Group B and A, respectively (*p* = 0.006) (Figure 4).

Among all the surgeries, 424 (7.6%) were performed as emergency procedures in Group A, whose number significantly increased from 315 in Group C (+34.6%) and 338 in Group B (+25.4%).

## Discussion

Early in the outbreak period of 2020, organ and tissue procurement and transplantation were dramatically affected by the COVID-19 pandemic in the United States and Europe. This was due to the lack of information on the risk of SARS-CoV-2 transmission from donor tissue to recipient. Indeed, no information was available on the effects of the COVID-19 infection on transplanted patients. After the initial deferral of elective surgical activity, the transplant rate began to increase, encouraged by local governments and in agreement with the national transplant Societies (4). At the beginning of the pandemic, an European multinational survey involving 64 eye banks showed a significant reduction in the number of corneas procured and distributed per month from March to May 2020, compared with the two previous years. Despite a wide variation in the international stringency of recommendations for corneal donor screening, negative correlation between the decrease in procurement and stringency were found (5).

Due to COVID-19, eye banks have globally developed different guidelines and criteria for evaluating the viability of donor corneas (6). The European Eye Bank Association (EEBA) has developed its own guidelines: first PCR test is required; if the PCR test is positive 14 days prior to death, the donor is ineligible; in addition, a nasopharyngeal swab postmortem is performed. However, individual risk assessment is necessary to assess the eligibility of the donor (7). The level of precautions adopted in the various countries ranged from automatic exclusion to retrieval if nasopharyngeal swab was negative in three categories of patients: (1) suspected to have COVID-19 but died from another cause; (2) recovered from COVID-19 and died later on from another cause; (3) asymptomatic at risk (5). For suspected donors, in European countries with the least stringent recommendations on retrieval tissues, the guidelines imposed a 14-day quarantine interval between recovery and death or between last symptom and death. The Countries with the most stringent donor guidelines, such as Austria, Belgium, France, Spain, imposed a prolonged period of up to 28 days free interval between recovery and death or between last symptom and death. No country made serology testing mandatory, but Italy recommended to be performed for epidemiologic reasons (5).

In Italy, one of the nations with the highest cornea procurement, the selection criteria released by the competent health Authority and therefore adopted by SIBO for donor screening, are summarized in Table 2.

**Table 2:** Italian screening criteria COVID-19 donor screening.

**Donor status**	**Recommendations for donor screening**
History of COVID-19/symptoms compatible with COVID-19 but swab not performed, or negative, or serological antibody to SARS-COVID- 2	Tissues should be collected 14 days after a documented virological recovery (resolution of symptoms and/or negative swab), and following a nasopharyngeal (genetic) swab for SARS-CoV-2 performed within 24 h of death^*^
History of close contact with COVID-19 patients (absence of clinical symptoms or with negative nasopharyngeal swab)	Tissues could be collected 14 days after the last close contact and following a nasopharyngeal (genetic) swab performed within 24 h of death, with negative results available before tissue distribution^*^.
Vaccinated	No contraindication

* *or, where present, a nasopharyngeal swab performed within 48 h prior to collection*.

For all the corneas collected by the eye banks, the use of 5% povidone-iodine (PVP–I) disinfection protocol was recommended during their retrieval. This has been shown to be effective at inactivating SARS-CoV-2 (as well as other viruses) (8).

The findings of this study confirmed that the COVID-19 pandemic partially reduced the number of cornea donations in comparison with the two previous years. From our data, which also analyzed the months following the lockdown, a reduction of 38 and 20% of corneal procurement and distributed tissues respectively emerged, compared to the 2019 and 2018.

The number of procured corneas represent, in our opinion, one of the main indexes of Eye Bank activity throughout each country. In this context, the apparent reduction in the number of retrieved corneas (more than 30%) during the last 12 months, compared to the previous 2 years, poses a number of considerations. In fact, this proportion appeared substantially contracted when compared to the one reported by the SIBO group, which presented a nearly 60% reduction in the number of retrieved corneas during the Italian lockdown period (3). Nonetheless, a nearly 30% reduction in a 1-year estimate of working activity might simply be related to the first 3-month of lockdown period, due to a dramatic reduction of surgical activity.

Early in the pandemic, before the introduction of Italian donor screening guidelines to exclude tissues potentially infected by SARS-CoV-2, an increase in discarded corneas considered at risk of contamination was reported (3). Current evidence indicates that SARS-CoV-2 transmission via corneal transplants from an infected donor to a healthy recipient host is a very rare event (9). Though, many studies speculate that the eye may be a possible entry route for SARS-Cov2, due to co-expression of SARS-CoV-2 entry receptors (ACE2 and TMPRSS2); the risk of transmission associated with cornea transplantation is very low. Therefore, the benefit of decedent testing would appear to be negligible (10, 11). In our opinion, the stringent screening and the proper manipulation of corneal tissues according to the guidelines provided by the competent health regulatory Authorities appear to be sufficient to prevent the transmission of SARS-CoV-2 to cornea transplant recipients.

During the pandemic a substantial reduction in overall eye surgical procedures were reported in the first 2 months of pandemic corresponding to the Italian national lockdown (10 March−9 May 2020). The reduction included predominantly elective surgeries. However, urgent procedures and intravitreal injections were also heavily affected (12). The deferral of elective surgery resulted in a significant increase in expired tissues to be used for corneal transplants compared to the previous 2 years. Against a relative reduction in the procurement and the distribution, the percentage of the transplants carried out during the “annus horribilis” was not dramatically affected. During the first 3 months of the COVID-19 lockdown period, the number of performed transplants both PK and EK were reduced by more than 50% compared to the same months of the previous 2 years (3). In our report, the number of PKs decreased of 29 and 33% compared to 2019 and 2018 respectively. The ALKs reduced of 25 and 14% compared to group B and C, respectively. Instead, between the EKs, the DMEK increased slightly compared to previous years. The reduction in the number of PKs in Group A compared to the previous 2 years may have been only partially affected by the COVID-19 pandemic; on one hand, the introduction of lamellar keratoplasty techniques has led to a general decrease in the percentage of PKs in recent years (13); on the other hand, the concomitant reduction of ALKs, although less than the PKs, could be justified by the suspension or deferral of elective surgical activity and by the delay in the treatment of pathologies that during the lockdown have been underestimated as reported for the rhegmatogenous retinal detachment (12). The relative increase of EKs, even during the pandemic, confirms the trend of recent years, with a progressive increase in endothelial selective corneal transplants and can be explained by the higher likelihood of this procedure being performed under local anesthesia and not requiring access to anesthetic facilities (14, 15). A recent report from India confirms that there was a gradual and incremental increase in all types of keratoplasties in the unlock phase, which exceeded the preceding years' monthly numbers in February and March 2021 (16).

Despite the difficult period, in relative terms, transplant activities have not suffered a profound negative impact. The possible explanation for this positive report may depend both on the guidelines adopted by eye bank that helped to improve screening by avoiding a loss of tissue, and on the competent authorities that encouraged transplantation activity. Therefore, the whole system from eye banks to surgery has held up well, and the advance in corneal transplantation has gone ahead.

## Conclusions

In conclusion, while the lockdown-related restrictions caused tremendous impact on eye banking/corneal transplant organization, our data support the hypothesis of an efficacious reaction with a constant and vigorous working activity resumption. Moreover, the increase in endothelial keratoplasty surgery, despite the pandemic, is a sign that corneal transplant surgery continues to evolve.

## Italian Society Eye Bank (SIBO) Group

Imola: Paola Bonci; Cosenza: Giuseppe Calabrò; Pavia: Roberto Ceccuzzi; Fabriano: Massimiliano Corneli; L'Aquila: Germano Genitti; Lucca: Claudio Giannarini; Monza: Raffaela Mistò; Rome: Augusto Pocobelli; Naples: Achille Tortori; Genoa: Davide Venzano.

## Data Availability Statement

The raw data supporting the conclusions of this article will be made available by the authors, without undue reservation.

## Author Contributions

RM, MC, FG, DP, and FA: conceptualization. RM, MC, FA, FG, GP, MB, and CN: methodology. FA, GP, and CN: formal analysis. RM, MC, FA, MB, GP, and DP: investigation. RM, MC, FA, FG, GP, and DP: data curation. RM, MC, FA, and DP: writing-original draft preparation and writing-review and editing. All authors contributed to the article and approved the submitted version.

## Conflict of Interest

The authors declare that the research was conducted in the absence of any commercial or financial relationships that could be construed as a potential conflict of interest.

## Publisher's Note

All claims expressed in this article are solely those of the authors and do not necessarily represent those of their affiliated organizations, or those of the publisher, the editors and the reviewers. Any product that may be evaluated in this article, or claim that may be made by its manufacturer, is not guaranteed or endorsed by the publisher.
